# Strap muscle invasion in differentiated thyroid cancer does not impact disease-specific survival: a population-based study

**DOI:** 10.1038/s41598-020-75161-y

**Published:** 2020-10-26

**Authors:** Ja Kyung Yoon, Jandee Lee, Eun-Kyung Kim, Jung Hyun Yoon, Vivian Youngjean Park, Kyunghwa Han, Jin Young Kwak

**Affiliations:** 1 Department of Radiology and Research Institute of Radiological Science, Severance Hospital, Yonsei University College of Medicine, 50-1 Yonsei-ro, Seodaemun-gu, Seoul, 03722 South Korea; 2 Department of Surgery, Severance Hospital, Yonsei Cancer Center, Yonsei University College of Medicine, Seoul, South Korea; 3 Center for Clinical Imaging Data Science, Severance Hospital, Yonsei University College of Medicine, 50-1 Yonsei-ro, Seodaemun-gu, Seoul, 03722 South Korea

**Keywords:** Thyroid cancer, Cancer

## Abstract

The American Joint Committee on Cancer (AJCC) 8^th^ TNM staging system of differentiated thyroid cancer defines gross strap muscle invasion as T3b stage. However, the impact of strap muscle invasion on disease-specific survival (DSS) remains controversial. To elucidate the survival impact of strap muscle invasion of any degree in thyroid cancers, the Surveillance, Epidemiology, and End Results (SEER) database (1973–2018) was queried for thyroid cancer only patients on July 2019 (n = 19,914). The Cox proportional hazard analysis with multivariable adjustment revealed that strap muscle invasion was not a significant factor for DSS in tumors equal to or smaller than 40 mm (hazard ratio (HR) = 1.620 [confidence interval (CI) 0.917 – 2.860]; *p* = 0.097). The competing risk analysis with multivariable adjustment showed that strap muscle invasion did not significantly impact DSS regardless of tumor size or cause of death (cancer-caused death (Subdistribution HR (SDHR) = 1.567 [CI 0.984 – 2.495]; *p* = 0.059); deaths to other causes (SDHR = 1.155 [CI 0.842 – 1.585]; *p* = 0.370). A “modified” staging schema discarding strap muscle invasion as a T stage criterion showed better 10-year DSS distinction between T stages. The modified staging schema may better reflect cancer-caused death risk and may prevent potential overstaging.

## Introduction

Extrathyroidal extension (ETE) is defined as the direct extension of the primary thyroid cancer into the adjacent structures, including strap muscles, trachea, larynx, esophagus, recurrent laryngeal nerve and/or major vasculature^1,2^. While strap muscle invasion was subclassified as minimal or gross, the American Joint Committee on Cancer (AJCC) 8th edition TNM staging schema discarded the minimal ETE category entirely while defining gross strap muscle invasion alone as the T3b stage. Validation studies have corroborated the new staging system by showing better allocation of relatively low-risk patients into lower stages with excellent survival outcomes^3^, and high-risk patients into more advanced stages^3–7^.

The newly introduced staging factor of gross strap muscle invasion alone (T3b) has been associated with higher risk of positive resection margin, bigger tumor size, higher recurrence rate, and lower disease survival rate^8–10^. However, studies have reported that strap muscle invasion alone, both minimal and gross, may not have significant influence on overall survival^11,12^, particularly in tumors equal to or smaller than 40 mm^13^. A recent study has also suggested similar disease-free survival rate between the minimal ETE and gross strap muscle invasion alone groups in papillary thyroid carcinomas with tumor size 10- to 40-mm^14^. With advances in surgical techniques and adjuvant therapies^13^, it is possible that tumors with strap muscle invasion alone may indeed have better prognosis than previously anticipated. Therefore, the impact of strap muscle invasion alone, both minimal and gross, on the survival of thyroid cancer patients needs to be elucidated to accurately allocate patients into appropriate staging groups according to individual risk, which will ensure longer disease-free survival and prevent overtreatment.

The Surveillance, Epidemiology, and End Results (SEER) database of the National Cancer Institute (NCI) collects cancer incidence and survival information from population-based cancer registries encompassing nearly 34.6% of the total population of the United States^15^. The SEER program is regarded as a critical database for cancer survival surveillance in the United States. This openly available database has compiled cancer incidence and survival data since 1973. Accordingly, it is an ideal database for analyzing the effect of gross ETE on overall survival in thyroid cancer.

Therefore, the purpose of this study was to explore the impact of strap muscle invasion alone, both minimal and gross, on the disease-specific survival (DSS) of differentiated thyroid cancer (DTC) patients using the SEER database, and to potentially improve the survival prediction power of the TNM staging schema.

## Results

### Baseline characteristics

Baseline patient demographics are presented in Table 1. The median age at diagnosis was 47.0 years old (interquartile range, 36.0–57.0 years old), with a median follow-up of 55.0 months (interquartile range, 32.0–85.0 months; range, 1.0–155.0 months). A total of 18,085 (90.8%) patients were diagnosed with papillary carcinoma and its variants, while 1406 (7.1%) patients were diagnosed with follicular carcinoma and its variants. Other pathologies included oxyphilic adenocarcinoma (n = 355), non-encapsulated sclerosing carcinoma (n = 64), and clear cell adenocarcinoma (n = 4). Most patients underwent total thyroidectomy (n = 16,874, 84.7%). Adjuvant radiation therapy was performed in over half of the patients (n = 10,165, 51.0%), which included radioactive iodine (RAI) (n = 9740), radioactive implants (n = 120), beam radiation (n = 195), a combination of beam radiation with implants or isotopes (n = 66), and radiation therapy not otherwise specified (n = 44). Adjuvant chemotherapy was performed in only 57 (0.3%) patients. The distribution of T stage according to the AJCC 8th staging criteria is shown in Table 2.

**Table 1:** Baseline patient demographics.

Patient characteristics	Value	
**Age at diagnosis**^a^	47	(36.0, 57.0)
< 55 years old	13,688	(68.7)
≥ 55 years old	6226	(31.3)
**Sex**		
Male	4658	(23.4)
Female	15,256	(76.6)
**Race**		
White	16,063	(80.7)
Black	1371	(6.9)
Other	2480	(12.4)
Tumor size (mm)^a^	15	(8.0, 26.0)
**Pathology according to ICD-O-3**		
Papillary carcinoma	18,085	(90.8)
Follicular carcinoma	1406	(7.1)
Other	423	(2.1)
Multifocal	8213	(41.2)
**Extrathyroidal extension (ETE)**		
No or minimal invasion	17,703	(88.9)
Strap muscle invasion alone	1552	(7.8)
Major organ invasion	464	(2.3)
Major vessel invasion	195	(1.0)
LN metastasis	4897	(24.6)
Distant metastasis	229	(1.1)
Total thyroidectomy	16,874	(84.7)
Radiation therapy	10,165	(51.0)
Chemotherapy	57	(0.3)
Follow-up months^a^	55	(32.0, 85.0)

Data are presented as numbers (%) unless indicated otherwise.

*ICD-O-3* International Classification of Diseases for Oncology, *LN* lymph node.

^a^Data are shown as medians (1st quartile, 3rd quartile).

**Table 2:** 10-year DSS and T stage distribution according to the AJCC 8th TNM staging schema or modified TNM staging schema (n = 19,914).

	n (%)		10-year DSS (%)	HR	95% CI	*p* value
**AJCC TNM8**^a^						**< 0.001**
T1a	6610	(33.2)	99.4	1	Reference	
T1b	5258	(26.4)	99.4	2.008	0.920–4.386	0.080
T2	4249	(21.3)	98.0	5.381	2.676–10.821	**< 0.001**
T3a	1586	(8.0)	93.1	19.144	9.685–37.839	**< 0.001**
T3b	1552	(7.8)	96.4	13.177	6.442–26.955	**< 0.001**
T4a	464	(2.3)	86.2	65.291	32.946–129.388	**< 0.001**
T4b	195	(1.0)	70.0	146.927	74.023–291.632	**< 0.001**
**Modified TNM**^b^						**< 0.001**
T1a	6848	(34.4)	99.4	1.000	Reference	
T1b	5801	(29.1)	99.3	1.900	0.957–3.772	0.067
T2	4755	(23.9)	97.8	4.810	2.599–8.904	**< 0.001**
T3	1851	(9.3)	92.6	16.802	9.232–30.579	**< 0.001**
T4a	464	(2.3)	86.2	52.082	28.137–96.408	**< 0.001**
T4b	195	(1.0)	70.0	117.249	63.230–217.418	**< 0.001**

Statistically significant values are shown in bold.

*DSS* disease-specific survival, *HR* hazard ratio, *CI* confidence interval.

^a^Staging according to the American Joint Committee on Cancer (AJCC) 8th edition of TNM classification.

^b^Staging according to the suggested modified TNM staging schema discarding the strap muscle invasion factor from the T staging criteria.

### Prognostic impact of strap muscle invasion alone on 10-year DSS

Cox proportional hazard analysis was performed to estimate the prognostic impact of strap muscle invasion on DSS in all patients (n = 19,914) and in the subgroup of patients with tumor size equal to or smaller than 40 mm (n = 17,837) (Table 3). Univariable and multivariable analyses revealed that strap muscle invasion alone as well as age, tumor size, major organ invasion, major vessel invasion, LN metastasis, distant metastasis, and chemotherapy were significant prognostic factors for DSS. Subgroup analysis of tumors equal to or smaller than 40 mm in size showed that although strap muscle invasion alone was a significant prognostic factor for DSS on univariable Cox regression analysis, it was not a significant prognostic factor for DSS on multivariable analysis.

**Table 3:** Cox proportional hazard analysis for disease-specific survival (DSS) according to the AJCC 8th TNM staging schema.

Variables	All patients (n = 19,914)				Tumor size ≤ 40 mm (n = 17,837)			
	Univariable		Multivariable		Univariable		Multivariable	
	HR (95% CI)	*p* value	HR (95% CI)	*p* value	HR (95% CI)	*p* value	HR (95% CI)	*p* value
Age	1.089 (1.079, 1.100)	**< 0.001**	1.068 (1.058, 1.078)	**< 0.001**	1.101 (1.086, 1.116)	**< 0.001**	1.084 (1.068, 1.099)	**< 0.001**
Male sex	2.396 (0.322, 0.541)	**< 0.001**	1.197 (0.636, 1.098)	0.196	1.949 (0.350, 0.752)	**< 0.001**	1.203 (0.563, 1.228)	0.354
**Race**		0.300				0.257		
White	Reference		–	–	Reference		–	–
Black	0.688 (1.453, 0.364)	0.250	–	–	0.624 (0.229, 1.698)	0.356	–	–
Others	1.192 (0.827, 1.717)	0.346	–	–	1.375 (0.839, 2.252)	0.206	–	–
**Primary tumor size**		**< 0.001**				**< 0.001**		
≤ 10 mm	Reference		Reference		Reference		Reference	
10 < size ≤ 20 mm	2.421 (1.315, 4.457)	**0.005**	2.021 (1.088, 3.756)	**0.026**	2.414 (1.311, 4.444)	**0.005**	1.676 (0.895, 3.137)	0.107
20 < size ≤ 40 mm	5.886 (3.365, 10.296)	**< 0.001**	3.821 (2.146, 6.803)	**< 0.001**	5.886 (3.365, 10.296)	**< 0.001**	3.821 (2.146, 6.803)	**< 0.001**
> 40 mm	25.546 (14.923, 43.732)	**< 0.001**	7.613 (4.276, 13.554)	**< 0.001**				
**ETE**		**< 0.001**				**< 0.001**		
No or minimal	Reference		Reference		Reference		Reference	
Strap muscle invasion only	3.278 (2.191, 4.904)	**< 0.001**	1.592 (1.044, 2.427)	**0.031**	3.580 (2.097, 6.112)	**< 0.001**	1.620 (0.917, 2.860)	0.097
Major organ invasion	16.228 (11.513, 22.875)	**< 0.001**	2.933 (1.925, 4.467)	**< 0.001**	15.010 (9.081, 24.810)	**< 0.001**	2.429 (1.327, 4.447)	**0.004**
Major vessel invasion	36.492 (25.812, 51.592)	**< 0.001**	4.284 (2.824, 6.500)	**< 0.001**	30.001 (17.083, 52.689)	**< 0.001**	6.195 (3.392, 11.316)	**< 0.001**
Multifocality	1.078 (0.713, 1.207)	0.576	–	–	1.158 (0.803, 1.670)	0.400	–	–
LN metastasis	3.628 (2.804, 4.693)	**< 0.001**	1.783 (1.307, 2.431)	**< 0.001**	3.379 (2.348, 4.863)	**< 0.001**	2.150 (1.406, 3.287)	**< 0.001**
Distant metastasis	50.060 (37.600, 66.663)	**< 0.001**	6.439 (4.590, 9.034)	**< 0.001**	76.920 (51.083, 115.810)	**< 0.001**	14.532 (9.232, 22.875)	**< 0.001**
Total thyroidectomy	1.124 (0.781, 1.617)	0.529	–	–	1.210 (0.714, 2.052)	0.479	–	–
Radiation therapy	2.486 (1.856, 3.330)	**< 0.001**	1.177 (0.864, 1.605)	0.301	2.804 (1.841, 4.270)	**< 0.001**	1.423 (0.907, 2.233)	0.125
Chemotherapy	30.070 (17.814, 50.755)	**< 0.001**	4.945 (2.719, 8.995)	**< 0.001**	16.706 (6.162, 45.297)	**< 0.001**	6.459 (2.231, 18.694)	**< 0.001**

Statistically significant values are shown in bold.

*DSS* disease-specific survival, *HR* hazards ratio, *CI* confidence interval, *ETE* extrathyroidal extension, *LN* lymph node.

### Competing risk analysis of cancer-caused deaths and deaths to other causes

On multivariable competing risk analysis, strap muscle invasion alone did not significantly impact death due to either cancer or other causes in all patients as well as in the subgroup of tumors equal to or smaller than 40 mm (Table 4), although it was a significant factor on univariable analysis (Supplementary Table S1). In addition, in the subgroup of patients 55 years old of age or older, multivariable competing risk analysis showed that strap muscle invasion only did not significantly impact death due to any cause, despite its significance on univariable competing risk analysis (Supplementary Table S2).

**Table 4:** Multivariable competing risk analysis of clinical and pathological variables for cancer–caused death according to the AJCC 8th TNM staging schema.

Variables	All (n = 19,914)				Tumor size ≤ 40 mm (n = 17,837)			
	Cancer–caused death (n = 233)		Death to other causes (n = 409)		Cancer–caused death (n = 116)		Death to other causes (n = 305)	
	SDHR (95% CI)	*p* value	SDHR (95% CI)	*p* value	SDHR (95% CI)	*p* value	SDHR (95% CI)	*p* value
Age	1.063 (1.053, 1.074)	**< 0.001**	1.085 (1.077, 1.094)	**< 0.001**	1.080 (1.064, 1.098)	**< 0.001**	1.088 (1.077, 1.098)	**< 0.001**
Male sex	1.124 (0.831, 1.520)	0.450	1.835 (1.499, 2.245)	**< 0.001**	1.168 (0.761, 1.793)	0.480	1.995 (1.580, 2.519)	**< 0.001**
**Primary tumor size**								
≤ 10 mm	Reference		Reference		Reference		Reference	
10 < size ≤ 20 mm	1.990 (1.060, 3.736)	**0.032**	1.219 (0.916, 1.621	0.170	1.632 (0.846, 3.149)	0.140	1.205 (0.903, 1.609)	0.210
20 < size ≤ 40 mm	3.736 (2.058, 6.784)	**< 0.001**	1.360 (1.024, 1.807)	**0.034**	2.692 (1.432, 5.061)	**0.002**	1.323 (0.990, 1.769)	0.059
> 40 mm	7.085 (3.810, 13.173)	**< 0.001**	1.951 (1.448, 2.628)	**< 0.001**	–	–	–	–
**ETE**								
No or minimal	Reference		Reference		Reference		Reference	
Strap muscle invasion only	1.567 (0.984, 2.495)	0.059	1.155 (0.842, 1.585)	0.370	1.588 (0.831, 3.035)	0.160	1.025 (0.676, 1.556)	0.910
Major organ invasion	2.823 (1.676, 4.753)	**< 0.001**	1.328 (0.872, 2.021)	0.190	2.720 (1.298, 5.702)	**0.008**	1.007 (0.531, 1.910)	0.980
Major vessel invasion	4.427 (2.632, 7.445)	**< 0.001**	0.921 (0.508, 1.667)	0.790	6.001 (3.104, 11.603)	**< 0.001**	1.343 (0.638, 2.827)	0.440
LN metastasis	1.730 (1.185, 2.526)	**0.004**	1.173 (0.926, 1.485)	0.190	2.027 (1.235, 3.327)	**0.005**	1.180 (0.880, 1.583)	0.270
Distant metastasis	5.962 (3.850, 9.234)	**< 0.001**	1.672 (1.032, 2.709)	**0.037**	13.190 (7.718, 22.542)	**< 0.001**	1.666 (1.848, 3.273)	0.140
Radiation therapy	1.268 (0.902, 1.782)	0.170	0.702 (0.570, 0.864)	**0.001**	1.534 (0.933, 2.520)	0.091	0.746 (0.582, 0.956)	**0.021**
Chemotherapy	4.506 (2.022, 10.041)	**< 0.001**	2.720 (0.978, 7.563)	0.055	5.661 (2.213, 14.481)	**< 0.001**	3.212 (0.856, 12.054)	0.084

Statistically significant values are shown in bold.

*DSS* disease-specific survival, *SDHR* subdistribution hazards ratio, *CI* confidence interval, *ETE* extrathyroidal extension, *LN* lymph node.

### 10-year DSS according to the T stages of the AJCC 8th TNM staging and the modified TNM staging schemas

**Figure 1:**
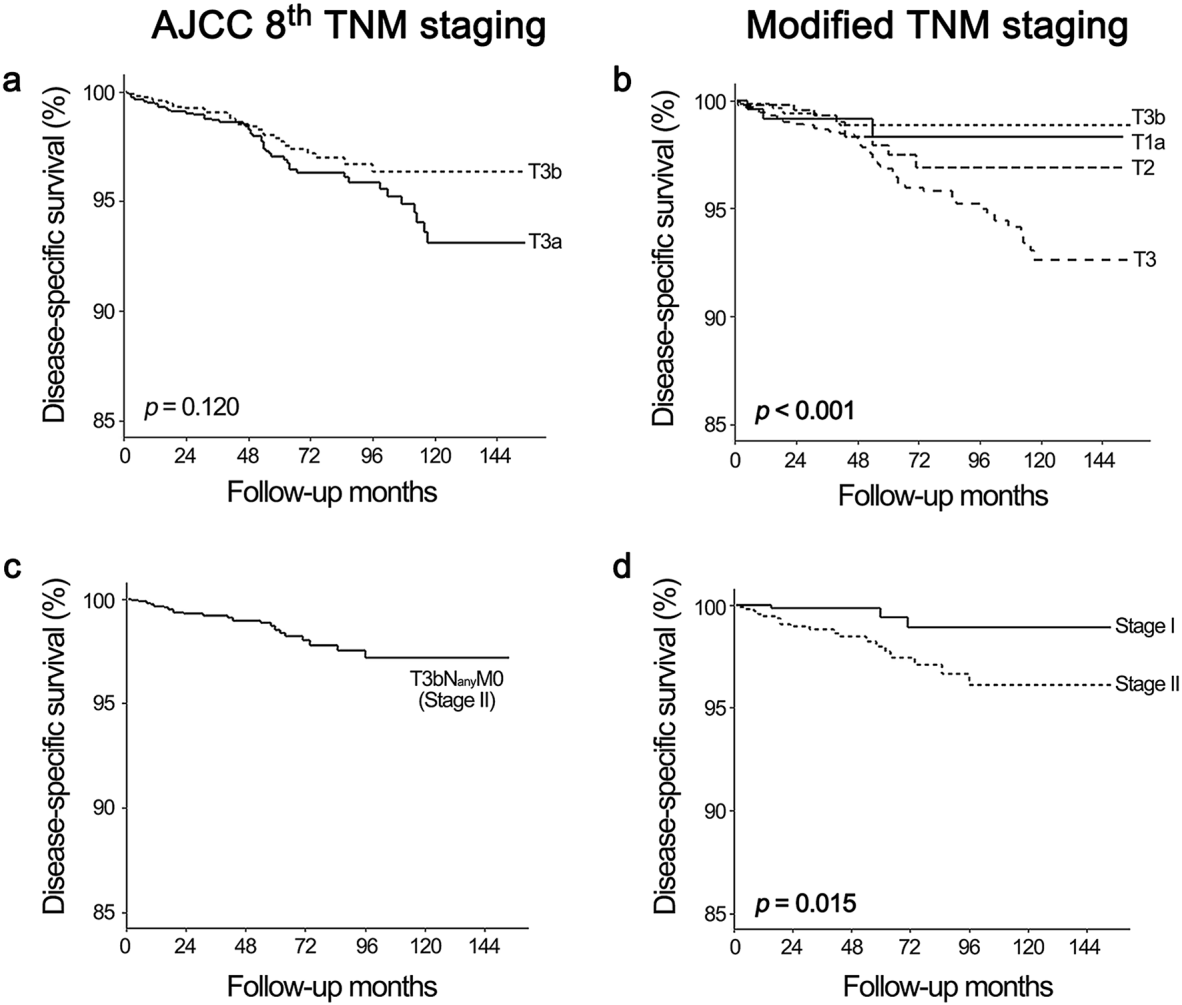
Comparison of DSS of the T3b subgroup according to the AJCC 8th TNM staging and modified TNM staging schemas and alluvial plot of restaging according to the two staging schemas. (**a**,**b**) Comparison of the disease-specific survival (DSS) between the T3a and T3b stages according to the AJCC 8th TNM staging schema (**a**) and the modified TNM staging schema (**b**). Reallocation of the T3a and T3b stages according to the modified TNM staging schema (**b**) showed significantly different DSS according to tumor size only (*p* < 0.001). (**c**,**d**) Comparison of DSS of the T3bN_any_M0 group according to the AJCC 8th TNM staging schema (**c**) and the modified TNM staging schema (**d**). The T3bN_any_M0 group (**c**) was classified as stage II with the AJCC 8th TNM staging schema. According to the modified TNM staging schema (**d**), the T3bN_any_M0 group was reallocated to either stage I or stage II with statistically significant difference in DSS (*p* = 0.015).

**Figure 2:**
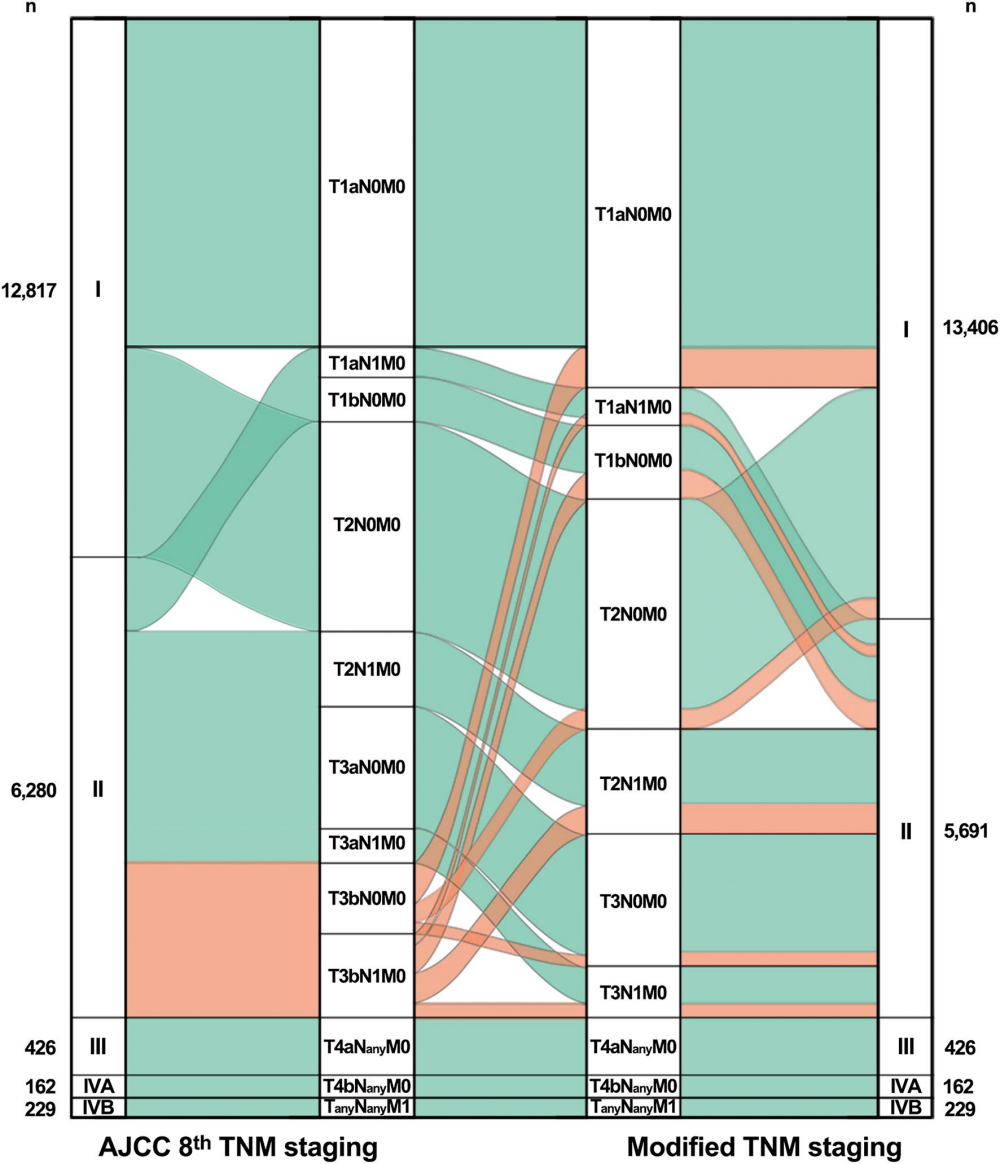
Restaging of the AJCC 8th TNM stages according to the modified TNM staging schema. The alluvial plot shows the reallocation flow for T stages and overall stages according to the modified TNM staging schema. Orange color indicates patients with strap muscle invasion (T3bN_any_M0) who were restaged according to tumor size. The number of patients in each T stage of the AJCC 8th TNM staging schema or the modified TNM staging schema are presented along the left and right margins.

Table 2 showed the 10-year DSS and the number of patients in each T stage according to either the AJCC 8th TNM staging schema or the “modified” TNM staging schema (discarding strap muscle invasion from the T stage criteria). While there was an overall negative correlation between T stage and 10-year DSS per the AJCC 8th staging schema, the T3b stage showed better 10-year DSS than the T3a stage (Fig. 1a). Restaging of the T3b stage (i.e., T3bN_any_M_any_) according to the modified TNM staging schema resulted in a total of 1552 patients assigned to T1a (n = 238, 15.3%), T1b (n = 543, 35.0%), T2 (n = 506, 32.6%), and T3 (n = 265, 17.1%) stages with significant difference in DSS (Fig. 1b). Among the 1518 patients without distant metastasis (i.e., T3bN_any_M0, stage II), 589 patients (38.8%) were downstaged to stage I, and there was significant difference in DSS after reallocation into either stage I or stage II (Fig. 1c,d). T stage and overall staging reallocation per the modified staging schema is shown in Fig. 2.

### Evaluation of the power of survival prediction for the AJCC 8th and the modified TNM staging schemas

The Harrell’s C concordance indices (C-indices) of the AJCC 8th and the modified TNM staging schemas was estimated as 0.9418 and 0.9405, respectively, but without statistically significant difference (*p* = 0.220). The proportion of variance explained (PVE) for DSS prediction with the AJCC 8th and the modified TNM staging schemas was estimated as 4.45% and 4.43%, respectively.

## Discussion

This study suggested that strap muscle invasion alone of any degree was not a statistically significant prognostic factor of DSS in all tumor sizes of DTC. In particular, this finding may significantly impact tumors equal to or smaller than 40 mm, which may be overstaged by the current AJCC 8th TNM staging schema. In addition, survival analysis of the T3b group according to the AJCC 8th TNM staging schema showed that it consists of a heterogeneous group of patients with significantly different DSS according to tumor size. T3b patients reallocated to either the T1 or T2 stage according to the suggested modified staging schema showed significantly better DSS compared to those in the T3 stage.

Historically, ETE was assumed to have a positive correlation with compromised survival in DTC^16,17^, but with some controversy^18,19^. While minimal ETE was totally discarded from the AJCC 8th TNM staging schema, gross strap muscle invasion was introduced as the new T3b stage regardless of tumor size. However, there have been suggestions that even gross strap muscle invasion does not impact survival in patients with DTC^8–11,13,20^. Indeed, the AJCC 8th staging schema contains in-stage heterogeneity in regards to DSS prediction^21^. One potential source of this heterogeneity may be due to the prognostic impact of strap muscle invasion. A recent study of a total of 2804 patients and demonstrated that perithyroidal soft tissue or strap muscle invasion showed disease-free survival, overall survival and DSS comparable to those of intrathyroidal tumors equal to or smaller than 40 mm (median follow-up 59 months; range, 12–192 months), although with small number of strap muscle invasion cases (n = 61)^13^. Another study on 3104 patients with either papillary or follicular thyroid carcinoma revealed that gross strap muscle invasion does not significantly impact DSS in tumors equal to or smaller than 40 mm, and that DSS of the T3b stage did not significantly differ from that of the T2 stage (median follow-up 10 years; interquartile range, 8.1–12 years)^11^.

This population-based study utilized the SEER database^15^ without discrimination of tumor size to reveal that strap muscle invasion alone does not significantly impact DSS, regardless of tumor size or cause of death. This may have particular clinical impact on tumors equal to or smaller than 40 mm which may be appropriately downstaged. Although the estimated C-indices and PVE did not reveal statistically significant differences in the predictive power of the current AJCC 8th and the suggested modified TNM staging schemas, better survival curve separation was observed per the modified staging schema with statistical significance.

Invasion of strap muscle in any degree may not have significant survival impact due to the relative complexity of posterior anatomical structures such as trachea, recurrent laryngeal nerve, and prevertebral fascia. Critical laryngotracheal structure invasion leads to a higher possibility of incomplete resection and therefore to higher clinical recurrence and poorer prognosis^9^. On the other hand, strap muscles can be relatively easily resected in the presence of tumor invasion^13,22^, accounting for their lack of significant impact on DSS. Consequently, anterior or posterior ETE have been suggested as more appropriate staging factors than gross strap muscle invasion^13,23^. Therefore, the dismissal of both minimal and gross strap muscle invasion may lead to better allocation by the TNM staging schema and better reflection of DSS, as suggested by the modified staging schema, particularly for tumors equal to or smaller than 40 mm.

There are several limitations to our study. First, the retrospective nature of this study may have caused an inherent bias. Second, the SEER database did not distinguish between minimal and gross strap muscle invasion. However, any ETE beyond strap muscle invasion was explicitly recorded and an inference was made that the code for strap muscle invasion included both minimal and gross strap muscle invasion collectively. Thus, the purpose of this study was limited to exploring the potential changes in TNM staging with the collective dismissal of both minimal and gross strap muscle invasions. Third, the SEER database did not include recurrence data, which limited the analysis of the impact of strap muscle invasion alone on disease-free survival. Strengths of the present study include the use of a national, comprehensive database with analyses of prognostic variables in all tumor size groups as well as tumor size and age subgroups. Moreover, competing risk analysis offered a more accurate evaluation of potential prognostic variables relative to other causes of death.

In conclusion, strap muscle invasion alone of any degree does not significantly impact DSS in DTC patients, regardless of tumor size. The modified TNM staging schema suggests potential modification of the TNM staging schema to better reflect the risk of cancer-caused death and to prevent potential overstaging of tumors, particularly those equal to or smaller than 40 mm in size.

## Methods

### SEER database and study population

**Figure 3:**
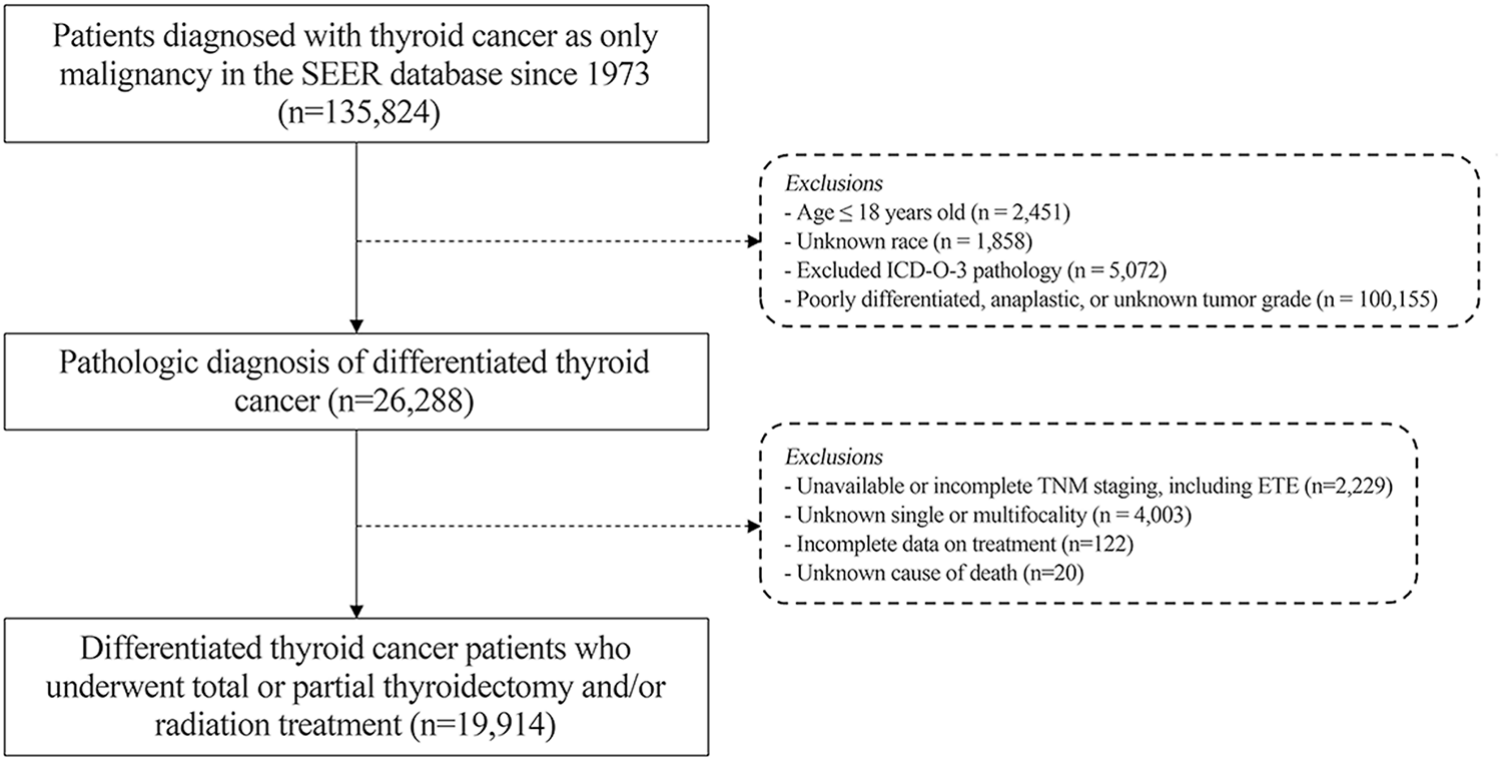
Flow diagram of the SEER database search. Flow diagram of the SEER database with inclusion and exclusion criteria.

The SEER database was accessed on July 4th, 2019, and queried for all patients diagnosed with DTC alone. Inclusion criteria were as follows: (1) patients older than 18 years old, (2) patients diagnosed with only DTC according to the International Classification of Disease for Oncology, third edition (ICD-O-3) codes (Supplementary Table S3), and (3) patients who underwent surgery with or without adjuvant radiation therapy or chemotherapy. Patients with medullary carcinoma, mixed medullary carcinoma component, insular carcinoma, and anaplastic thyroid cancer were excluded. In addition, patients with unknown race, unknown tumor grade, unknown ETE, unavailable or incomplete TNM staging data, death certificate or autopsy alone, or unknown cause of death were excluded (Fig. 3). The extension item of the SEER Cancer Schema (CS) version 02.05.50, which encodes tumor ETE based on pathologic and/or clinical information, does not explicitly distinguish between minimal or gross strap muscle invasion. However, since more advanced organ invasion (i.e., recurrent laryngeal nerve, vagus nerve, major vessels, major organs or prevertebral fascia) was clearly distinguished by the CS extension coding, the invasion of omohyoid, sternohyoid, sternothyroid and/or thyrohyoid muscles (code 450) was considered to include both minimal and gross strap muscle invasion collectively. This code was regarded as the T3b stage per the AJCC 8th TNM staging schema in this study.

A total of 19,914 patients were included in the final analysis of demographic variables including age at diagnosis, race, sex, year of diagnosis and survival status. Pathologic characteristics included single or multifocal disease, tumor size, ETE, LN metastasis and distant metastasis. This retrospective study utilizing a publicly available database with de-identified records did not require informed consent from the SEER registered cases, and Institutional Review Board approval was formally waived.

### Primary outcome

The primary outcome of this study was cancer-caused death from DTC. DSS was defined as the time from date of diagnosis until cancer-caused death or last censoring.

### Tumor staging

Because the currently available SEER database only provides TNM staging according to the AJCC 7th or previous staging schemas, the included 19,914 patients were restaged according to the AJCC 8th TNM staging schema using the recorded pathologic characteristics. Strap muscle invasion alone (code 450 of the CS extension item in the SEER CS version 02.05.50) was categorized as T3b stage, while pericapsular soft tissue or connective tissue invasion was categorized according to tumor size only. Major organ invasion including gross invasion of the subcutaneous soft tissue, larynx, trachea, esophagus, or recurrent laryngeal nerve from any tumor size was classified as T4a stage per the AJCC 8th TNM staging schema. Likewise, major vessel and/or prevertebral fascia invasions including the encasement of carotid artery or mediastinal vessels from any tumor size were classified as T4b stage.

Based on our hypothesis that minimal and gross strap muscle invasion collectively in the absence of other risk factors does not significantly impact DSS, we suggested a “modified” staging schema thereby discarding strap muscle invasion of any degree from the T staging criteria. Therefore, T3b stage according to the AJCC 8th TNM staging schema were reallocated depending on tumor size only, into either the T1a, T1b, T2, or T3 stage per the modified staging schema. The staging of major adjacent structures extension (i.e., T4a or T4b stages) remained unchanged.

### Statistical analysis

Statistical analyses were performed using commercially available software (R software, version 3.6.1 (https://www.R-project.org, R Foundation for Statistical Computing, Vienna, Austria) with the application of appropriate R packages (survival, survminer, cmprsk, compareC, ggplot2 and alluvial)^24–29^. The Cox proportional hazard analysis was used to examine the effects of clinical factors and histopathologic characteristics of DTC on DSS. Competing risk analysis by proportional subdistribution hazards regression modeling was performed^26,30^. In this study, competing risk was considered death due to other causes since deaths unrelated to DTC may obscure the ability to observe cancer-caused deaths^31^. Kaplan–Meier estimation and the log rank test were used to assess 10-year DSS probability and DSS curves according to T stages based on either the AJCC 8th TNM staging schema or the modified TNM staging schema. The measure of discrimination for survival prediction was estimated using C-index, and goodness-of-fit for survival prediction was estimated using the PVE for both staging schemas^32^. All tests were two-sided, and a *p*-value of less than 0.05 was considered statistically significant.

## Supplementary information

Supplementary Tables.
