# Supplementary material for: Strap muscle invasion in differentiated thyroid cancer does not impact disease-specific survival: a population-based study
Source: Sci Rep. 2020 Oct 26;10:18248. doi: 10.1038/s41598-020-75161-y (PMC7589560; doi:10.1038/s41598-020-75161-y)
Supplement: Supplementary file 1 — Supplementary Tables. [file 41598_2020_75161_MOESM1_ESM.docx]

**Strap muscle invasion in differentiated thyroid cancer does not impact disease-specific survival: a population-based study**

Ja Kyung Yoon^1^, Jandee Lee^2^, Eun-Kyung Kim^3^, Jung Hyun Yoon^3^, Vivian Youngjean Park^3^, Kyunghwa Han^3, 4,*^, Jin Young Kwak^3,*^

^1^Department of Radiology, Severance Hospital, Yonsei University College of Medicine, 50-1 Yonsei-ro, Seodaemungu, Seoul 03722, Korea

^2^Department of Surgery, Severance Hospital, Yonsei Cancer Center, Yonsei University College of Medicine, Seoul, South Korea

^3^Department of Radiology and Research Institute of Radiological Science, Yonsei University College of Medicine, 50-1 Yonsei-ro, Seodaemungu, Seoul 03722, Korea

^4^Center for Clinical Imaging Data Science, Severance Hospital, Yonsei University College of Medicine, 50-1 Yonsei-ro, Seodaemungu, Seoul 03722, Korea

^*^K.H. and J.Y.K. contributed equally to this work.

**Corresponding author 1**: Jin Young Kwak

Department of Radiology, Severance Hospital, Yonsei University, College of Medicine
50-1 Yonsei-ro Seodaemun-gu, Seoul 03722, Korea
Tel.: 82-2- 2228-7400, Fax.: 82-2-393-3035
E-mail: Jin Young Kwak (docjin@yuhs.ac)

**Corresponding author 2**: Kyunghwa Han

Department of Radiology and Research Institute of Radiological Science,
Center for Clinical Imaging Data Science
Severance Hospital, Yonsei University College of Medicine
50-1 Yonsei-ro, Seodaemungu, Seoul 03722, Korea

Tel.: 82-2- 2228-7400, Fax.: 82-2-393-3035
E-mail: Kyunghwa Han (khhan@yuhs.ac)

**Supplementary Table S1. Univariable competing risk analysis of clinical and pathological variables for cancer-caused death according to the AJCC 8^th^ TNM staging schema.**

| **Variables** | | **All (n=19,914)** | | | | |  | **Tumor size ≤40 mm (n=17,837)** | | | | |
| --- | --- | --- | --- | --- | --- | --- | --- | --- | --- | --- | --- | --- |
|  |  | **Cancer-caused death (n=233)** | |  | **Other caused death (n=409)** | |  | **Cancer-caused death (n=116)** | |  | **Other caused death (n=305)** | |
|  |  | **SDHR (95% CI)** | ***p* value** |  | **SDHR (95% CI)** | ***p* value** |  | **SDHR (95% CI)** | ***p* value** |  | **SDHR (95% CI)** | ***p* value** |
| Age | | 1.085 (1.075, 1.095) | **<0.001** |  | 1.092 (1.083, 1.100) | **<0.001** |  | 1.097 (1.081, 1.113) | **<0.001** |  | 1.091 (1.080, 1.101) | **<0.001** |
| Male sex | | 0.423 (0.326, 0.549) | **<0.001** |  | 0.404 (0.332, 0.492) | **<0.001** |  | 0.519 (0.354, 0.762) | **0.001** |  | 0.400 (0.318, 0.503) | **<0.001** |
| Race | |  |  |  |  |  |  |  |  |  |  |  |
|  | White | Reference |  |  | Reference |  |  | Reference |  |  | Reference |  |
|  | Black | 0.682 (0.361, 1.289) | 0.240 |  | 1.514 (1.082, 2.118) | **0.016** |  | 0.620 (0.228, 1.684) | 0.350 |  | 1.458 (0.966, 2.200) | 0.072 |
|  | Others | 1.198 (0.831, 1.725) | 0.330 |  | 0.647 (0.453, 0.924) | **0.017** |  | 1.381 (0.843, 2.263) | 0.200 |  | 0.602 (0.393, 0.922) | **0.020** |
| Primary tumor size | |  | **<0.001** |  |  |  |  |  |  |  |  |  |
|  | ≤ 10 mm | Reference |  |  | Reference |  |  | Reference |  |  | Reference |  |
|  | 10 < size ≤ 20 mm | 2.425 (1.315, 4.470) | **0.004** |  | 0.996 (0.755, 1.314) | 0.980 |  | 2.416 (1.309, 4.461) | **0.005** |  | 1.458 (0.966, 2.200) | 0.072 |
|  | 20 < size ≤ 40 mm | 5.883 (3.360, 10.301) | **<0.001** |  | 1.272 (0.970, 1.668) | 0.082 |  | 5.860 (3.342, 10.276) | **<0.001** |  | 0.602 (0.393, 0.922) | **0.020** |
|  | > 40 mm | 25.034 (14.608, 42.901) | **<0.001** |  | 3.098 (2.360, 4.068) | **<0.001** |  | - | - |  | - | - |
| ETE | |  |  |  |  |  |  |  |  |  |  |  |
|  | No or minimal | Reference |  |  | Reference |  |  | Reference |  |  | Reference |  |
|  | Strap muscle invasion only | 3.259 (2.180, 4.874) | **<0.001** |  | 1.162 (1.162-2.204) | **0.004** |  | 3.575 (2.096, 6.096) | **<0.001** |  | 1.253 (0.825, 1.901) | 0.290 |
|  | Major organ invasion | 15.734 (11.080, 22.344) | **<0.001** |  | 3.255 (2.216, 4.780) | **<0.001** |  | 14.800 (8.862, 24.718) | **<0.001** |  | 2.057 (1.146, 3.694 | **0.016** |
|  | Major vessel invasion | 35.019 (24.505, 50.043) | **<0.001** |  | 3.747 (2.246, 6.252) | **<0.001** |  | 29.079 (16.198, 52.206) | **<0.001** |  | 3.898 (1.994, 7.622) | **<0.001** |
| Multifocality | | 0.927 (0.712, 1.205) | 0.570 |  | 1.064 (0.875, 1.295) | 0.530 |  | 1.156 (0.803, 1.666) | 0.440 |  | 1.102 (0.879, 1.241) | **0.400** |
| LN metastasis | | 3.620 (2.799, 4.682) | **<0.001** |  | 1.083 (0.868, 1.352) | 0.480 |  | 3.378 (2.348, 4.861) | **<0.001** |  | 0.948 (0.725, 1.241) | 0.700 |
| Distant metastasis | | 45.281 (33.966, 60.364) | **<0.001** |  | 5.705 (3.721, 8.746) | **<0.001** |  | 71.143 (47.026, 107.629) | **<0.001** |  | 5.389 (2.854, 10.176) | **<0.001** |
| Total thyroidectomy | | 1.127 (0.782, 1.624) | 0.520 |  | 0.844 (0.658, 1.084) | 0.180 |  | 1.212 (0.713, 2.061) | 0.480 |  | 0.895 (0.667, 1.201) | 0.460 |
| Radiation therapy | | 2.499 (1.867, 3.343) | **<0.001** |  | 0.775 (0.639, 0.941) | **0.010** |  | 2.817 (1.854, 4.281) | **<0.001** |  | 0.764 (0.611, 0.956) | **0.019** |
| Chemotherapy | | 28.048 (15.819, 49.730) | **<0.001** |  | 4.320 (1.738, 10.737) | **0.002** |  | 15.919 (5.731, 44.219) | **<0.001** |  | 4.334 (1.342, 13.995) | **0.014** |

Statistically signiﬁcant values are shown in bold. *SDHR*, subdistribution hazard ratio; *CI*, confidence interval; *ETE*, extrathyroidal extension; *LN*, lymph node.

**Supplementary Table S2. Competing risk analysis of clinical and pathological variables for cancer-caused death according to the AJCC 8^th^ TNM staging schema in patients of 55 years of age or older (n=6,226).**

| **Variables** | | **Cancer-caused death (n=178)** | | | | |  | **Other caused death (n=308)** | | | | |
| --- | --- | --- | --- | --- | --- | --- | --- | --- | --- | --- | --- | --- |
|  |  | **Univariable** | |  | **Multivariable** | |  | **Univariable** | |  | **Multivariable** | |
|  |  | **SDHR (95% CI)** | ***p* value** |  | **SDHR (95% CI)** | ***p* value** |  | **SDHR (95% CI)** | ***p* value** |  | **SDHR (95% CI)** | ***p* value** |
| Age | | 1.080 (1.062, 1.098) | **<0.001** |  | 1.56 (1.036 – 1.076) | **<0.001** |  | 1.109 (1.096, 1.123) | **<0.001** |  | 1.102 (1.087 – 1.117) | **<0.001** |
| Male sex | | 0.614 (0.454, 0.831) | **0.002** |  | 0.949 (0.668 – 1.348) | 0.770 |  | 0.529 (0.421, 0.664) | **<0.001** |  | 1.770 (1.397 – 2.243) | **<0.001** |
| Race | |  |  |  |  |  |  |  |  |  |  |  |
|  | White | Reference |  |  | - | - |  | Reference |  |  | - | - |
|  | Black | 0.610 (0.286, 1.301) | 0.200 |  | - | - |  | 1.291 (0.852, 1.956) | 0.230 |  | - | - |
|  | Others | 1.578 (1.059, 2.352) | 0.025 |  | - | - |  | 0.802 (0.541, 1.189) | 0.270 |  | - | - |
| Primary tumor size | |  |  |  |  |  |  |  |  |  |  |  |
|  | ≤ 10 mm | Reference |  |  | Reference |  |  | Reference |  |  | Reference |  |
|  | 10 < size ≤ 20 mm | 2.800 (1.445, 5.426) | **0.002** |  | 1.963 (0.985 – 3.912) | 0.055 |  | 1.206 (0.860, 1.692) | 0.280 |  | 1.243 (0.876 – 1.763) | 0.220 |
|  | 20 < size ≤ 40 mm | 6.076 (3.301, 11.184) | **<0.001** |  | 3.086 (1.583 – 6.018) | **0.001** |  | 1.791 (1.302, 2.462) | **<0.001** |  | 1.549 (1.106 – 2.169) | **0.011** |
|  | > 40 mm | 18.387 (10.244, 33.005) | **<0.001** |  | 5.004 (2.484 – 10.082) | **<0.001** |  | 3.245 (2.363, 4.457) | **<0.001** |  | 2.004 (1.408 – 2.851) | **<0.001** |
| ETE | |  |  |  |  |  |  |  |  |  |  |  |
|  | No or minimal | Reference |  |  | Reference |  |  | Reference |  |  | Reference |  |
|  | Strap muscle invasion only | 2.914 (1.821, 4.662) | **<0.001** |  | 1.447 (0.835 – 2.508) | 0.190 |  | 1.449 (1.040, 2.162) | **0.030** |  | 1.121 (0.780 – 1.610) | 0.540 |
|  | Major organ invasion | 10.724 (7.137, 16.112) | **<0.001** |  | 2.411 (1.314 – 4.422) | **0.004** |  | 2.496 (1.624, 3.839) | **<0.001** |  | 1.260 (0.784 – 2.026) | 0.340 |
|  | Major vessel invasion | 20.175 (13.533, 30.078) | **<0.001** |  | 4.202 (2.372 – 7.444) | **<0.001** |  | 1.941 (1.095, 3.441) | **0.023** |  | 0.760 (0.391 – 1.477) | 0.420 |
| Multifocality | | 1.065 (0.789, 1.438) | 0.68 |  | - | - |  | 1.126 (0.896, 1.414) | 0.310 |  | - | - |
| LN metastasis | | 5.672 (4.216, 7.631) | **<0.001** |  | 2.061 (1.310 – 3.243) | **0.002** |  | 1.427 (1.093, 1.865) | **0.009** |  | 1.067 (0.799 – 1.425) | 0.660 |
| Distant metastasis | | 22.594 (16.400, 31.129) | **<0.001** |  | 5.957 (3.754 – 9.454) | **<0.001** |  | 2.836 (1.788, 4.498) | **<0.001** |  | 1.659 (0.998 – 2.759) | 0.051 |
| Total thyroidectomy | | 1.226 (0.812, 1.851) | 0.330 |  | - | - |  | 0.936 (0.702, 1.248) | 0.650 |  | - | - |
| Radiation therapy | | 2.564 (1.852, 3.550) | **<0.001** |  | 1.105 (0.749 – 1.630) | 0.610 |  | 0.872 (0.696, 1.093) | 0.230 |  | 0.704 (0.552 – 0.899) | **0.005** |
| Chemotherapy | | 16.675 (8.446, 32.921) | **<0.001** |  | 3.445 (1.344 – 8.828) | **0.010** |  | 3.128 (1.106, 8.849) | **0.032** |  | 3.295 (1.038 – 10.463) | **0.043** |

Statistically signiﬁcant values are shown in bold. *SDHR*, subdistribution hazard ratio; *CI*, confidence interval; *ETE*, extrathyroidal extension; *LN*, lymph node.

**Supplementary Table S3. Included histopathologic diagnosis of DTC according to ICD-O-3.**

| ICD-O-3 Histology/behavior code |  | Histopathology |
| --- | --- | --- |
| 8050/3 |  | Papillary carcinoma, NOS |
| 8260/3 |  | Papillary adenocarcinoma, NOS |
| 8330/3 |  | Follicular adenocarcinoma, NOS |
| 8331/3 |  | Follicular adenocarcinoma well diff. |
| 8332/3 |  | Follicular adenocarcinoma trabecular |
| 8333/3 |  | Fetal adenocarcinoma |
| 8335/3 |  | Follicular carcinoma, minimally invasive |
| 8339/3 |  | Follicular thyroid carcinoma (FTC), encapsulated angioinvasive |
| 8340/3 |  | Papillary carcinoma, follicular variant |
| 8341/3 |  | Papillary microcarcinoma |
| 8342/3 |  | Papillary carcinoma, oxyphilic cell |
| 8343/3 |  | Papillary carcinoma, encapsulated |
| 8344/3 |  | Papillary carcinoma, columnar cell |
| 8350/3 |  | Nonencapsulated sclerosing carcinoma |
| 8290/3 |  | Oxyphilic adenocarcinoma |
| 8310/3 |  | Clear cell adenocarcinoma, NOS |
| 8450/3 |  | Papillary cystadenocarcinoma, NOS |

*ICD-O-3*, International Classification of Diseases for Oncology; *NOS*, not otherwise specified.
